# Paradoxical Use of Benralizumab in Reactive Hypereosinophilia from Toxocariasis and Tuberculosis Co-Infection—Case Report and Literature Review

**DOI:** 10.3390/ijms26178117

**Published:** 2025-08-22

**Authors:** Nicoleta Sorina Bertici, Talida Georgiana Cut, Amalia Ridichie, Andrei Raul Manzur, Razvan Adrian Bertici

**Affiliations:** 1 Division of Pulmonology, Department of Infectious Diseases, “Victor Babeș” University of Medicine and Pharmacy Timișoara, Eftimie Murgu Square No. 2, 300041 Timișoara, Romania; bertici.nicoleta@umft.ro; 2 Center for Research and Innovation in Personalised Medicine of Respiratory Diseases, Pulmonology University Clinic, “Victor Babeș” University of Medicine and Pharmacy Timișoara, Eftimie Murgu Square No. 2, 300041 Timișoara, Romania; razvan.bertici@umft.ro; 3 IInd Pulmonology Ward, Clinical Hospital of Infectious Diseases and Pulmonology “Victor Babeș” Timișoara, Gheorghe Adam Street 13, 300310 Timișoara, Romania; 4 Division of Infectious Diseases, Department of Infectious Diseases, “Victor Babeș” University of Medicine and Pharmacy Timișoara, Eftimie Murgu Square No. 2, 300041 Timișoara, Romania; 5 Center for Ethics in Human Genetic Identifications, “Victor Babeș” University of Medicine and Pharmacy Timișoara, Eftimie Murgu Square No. 2, 300041 Timișoara, Romania; 6 Advanced Instrumental Screening Center, Faculty of Pharmacy, “Victor Babeş” University of Medicine and Pharmacy Timișoara, Eftimie Murgu Square No. 2, 300041 Timișoara, Romania; amalia.ridichie@umft.ro; 7 Faculty of Chemical Engineering Biotechnologies and Environmental Protection, University Politehnica Timisoara, 6 Vasile Parvan Boulevard, 300223 Timisoara, Romania; 8 Doctoral School Medicine-Pharmacy, “Victor Babeș” University of Medicine and Pharmacy Timișoara, Eftimie Murgu Square No. 2, 300041 Timișoara, Romania; andrei.manzur@umft.ro; 9 Institute for Cardiovascular Diseases of Timișoara, “Victor Babeș” University of Medicine and Pharmacy Timișoara, Gheorghe Adam Street, No. 13A, 300310 Timișoara, Romania; 10 First Department of Neurology, “Pius Brînzeu” Emergency County Hospital, Liviu Rebreanu Avenue No. 156, 300736 Timișoara, Romania

**Keywords:** toxocariasis, tuberculosis, hypereosinophilia, asthma, benralizumab

## Abstract

Tuberculosis and parasitic infections, including *Toxocara*, frequently coexist in many regions worldwide, yet their interaction remains poorly understood. Tuberculosis triggers a type 1 immune response characterized by IL-12, IFN-γ, and TNF-α production, while toxocariasis elicits a type 2 response, mediated by cytokines such as IL-4, IL-5, IL-13, and IL-33. The coexistence of these divergent immune pathways can disrupt immune regulation and impair the host’s ability to control both infections, potentially leading to persistent hypereosinophilia. We illustrate this complex interplay through a real-world case involving a heavy smoker in whom *Toxocara* infection likely reactivated latent tuberculosis, resulting in severe, unexplained hypereosinophilia and late-onset asthma with recurrent exacerbations. After excluding other causes and completing full antituberculosis therapy along with three courses of antiparasitic treatment and systemic corticosteroids, hypereosinophilia persisted. The introduction of benralizumab, a biologic therapy targeting IL-5Rα, led to a rapid reduction in eosinophils to normal ranges and significant clinical improvement. This case underscores the diagnostic and therapeutic challenges posed by the intersection of common infections and highlights that even a neglected parasitic infection such as toxocariasis can underlie severe respiratory complications with eosinophilia, where paradoxically biologic therapy may ultimately provide a very effective intervention.

## 1. Introduction

Human toxocariasis is a neglected parasitic disease of global importance, with 19% worldwide seroprevalence [1], and with estimated ~1.4 billion people worldwide infected, particularly in subtropical and tropical regions [2]. The infection is usually asymptomatic, rarely diagnosed, often mimicking other conditions [3]. Humans become infected by accidental ingestion of *Toxocara canis* eggs present in contaminated food, water, or soil. After ingestion, the larvae hatch in the intestine and can develop atypical clinical syndromes, either by reaching different tissues (liver, lung, brain) or through an induced immune response [4]. Helminth infections usually trigger a type 2 immune response in the host, by releasing cytokines such as interleukins (IL): IL-4, IL-5, IL-13 and IL-33 [5,6,7]. Recent studies demonstrated that the IL-33/ST2 pathway is related to parasite burden on the liver and brain and increases the number of eosinophils in the blood and tissues. In addition, it involved pulmonary immune response and granulomas with impact in lung function [8,9].

Regarding tuberculosis, it is as old as humanity itself and WHO’s Global tuberculosis report for 2024 reveals that it has probably returned to being the world’s leading infectious disease killer. In 2023, a global total of 8.2 million people were reported as newly diagnosed with tuberculosis and 1.25 million deaths [10].

Hypereosinophilia (HE) has generally been defined as a peripheral blood eosinophil count greater than 1.5 × 109/L and it can be episodic, transient, or persistent [11,12,13]. The temporal interval to define “persistent” eosinophilia has been widely discussed. In the first definition comprised in Chusid criteria, an absolute eosinophil count (AEC) persistently increased longer than 6 months above 1.5 × 109/L was required [14]. Then, a 4-week interval was proposed [15]. Finally, the latest Working Conference on Eosinophil Disorders and Syndromes (Vienna, 24–26 September 2021) defined “persistent” HE as the evidence of AEC ≥ 1.5 × 109/L recorded at least twice in a minimum time interval of two weeks. HE can be primary or often secondary (reactive) and has numerous causes that require multidisciplinary diagnostic evaluation [16]. In developing countries, eosinophilia most commonly derives from infections, particularly tissue-invasive parasites [17].

Hypereosinophilic syndrome (HES) is a rare hematologic disorder without a known cause (is mainly a diagnosis of exclusion, after ruling out all other etiological conditions) [18] characterized by the overproduction of eosinophils in the bone marrow and resulting in high blood eosinophil levels [19,20]. The incidence and prevalence of HES are not well characterized, but a study from primary care in United Kingdom published in 2021 showed an increasing trend in both incidence and prevalence of HES between 2010 and 2018, although HES remains a very rare condition. Thus, the incidence of HES ranged from less than 0.04, 95% confidence interval (CI) (0.01–0.07) to 0.17, 95% CI (0.10–0.26) per 100,000 person-years and the prevalence ranged from 0.15, 95% CI (0.10–0.25) to 0.89, 95% CI (0.74–1.09) cases per 100,000 persons [21].

The objective of this article is to highlight and describe clinical manifestations, diagnostic approach and treatment options in cases of concomitant tuberculosis and toxocariasis, two infections that are frequently encountered in clinical practice.

## 2. Results

**Figure 1:**
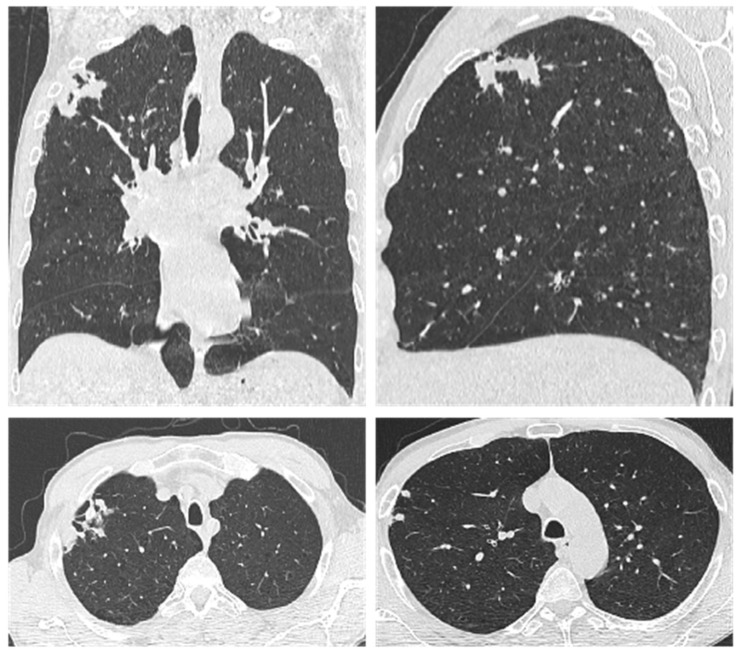
Chest CT from 13 January 2023 showed isolated and confluent nodular and micronodular opacities, some with pleural connection and included microcalcifications, located in the apical and anterior segment of the right upper lobe, bilateral centrilobular pulmonary emphysematous dystrophy, predominantly in the upper lobes.

A 55-year-old male patient from a rural area, unemployed and receiving only social assistance from the local municipality, with a significant smoking history (30 pack-years), was first diagnosed in January 2023 with chronic obstructive pulmonary disease (COPD) of moderate form, stage II, and risk class B, and fibronodular sequelae in the upper lobe of the right lung, most likely of post-tuberculous origin. A chest computed tomography (CT) scan performed on 13 January 2023 (Figure 1) revealed isolated and confluent nodular and micronodular opacities, some with pleural contact and microcalcifications, located in the apical and anterior segments of the right upper lobe. In addition, bilateral centrilobular emphysematous dystrophy was noted, predominantly in the upper lobes. The Quantiferon TB GOLD Plus test was positive at that time, but all bacteriological investigations for tuberculosis, including the GeneXpert molecular test, were negative. Also, the blood count was within normal limits. Background bronchodilator therapy was initiated with a dual combination of long-acting muscarinic antagonists (LAMA) and long-acting beta2 agonists (LABA), with Salbutamol used as needed. The patient showed a favorable clinical evolution, although chronic cough, intermittent morning sputum, and exertional dyspnea on moderate effort persisted, symptoms primarily attributed to COPD.

**Figure 2:**
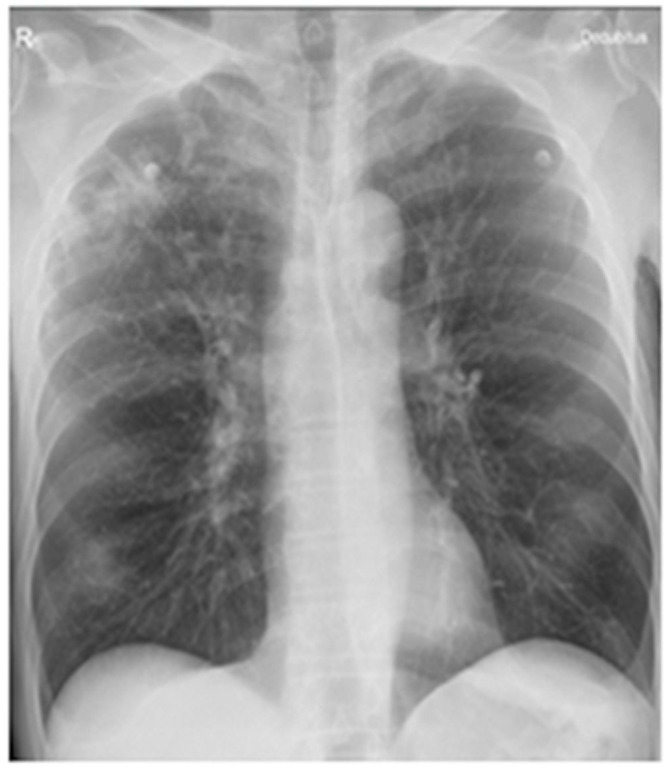
Chest X-ray from 17 December 2023, shows new external subclavicular nodular infiltrative lesions in the right lung and multiple diffusely defined ground-glass opacities in both lung bases.

After ten months, on 16 December 2023, the patient presented to the emergency department with resting dyspnea, wheezing, severe dry cough, headache, vertigo, loss of appetite, and marked asthenia. Clinical examination revealed underweight, pallor of the skin, and bilateral disseminated bronchial rales. A chest X-ray identified external subclavicular nodular infiltrative lesions on the right side and diffusely demarcated ground-glass opacities in both lung bases (Figure 2).

The bacteriological examination of the sputum sample for *Mycobacterium tuberculosis* yielded negative results on direct smear microscopy employing acid-fast bacilli (AFB) staining. However, the GeneXpert MTB/RIF assay, an automated nucleic acid amplification test that detects the DNA of *Mycobacterium tuberculosis* and mutations in the rpoB gene was positive, indicating a low bacterial load with no evidence of drug resistance. Consequently, a diagnosis of active pulmonary tuberculosis was established based on the following elements: male, heavy smoker, from an endemic area, symptomatic, with dynamically evolving lung lesions and with the GeneXpert test going from negative to positive. Standard antituberculous therapy was initiated with daily administration of Isoniazid, Rifampicin, Pyrazinamide, Ethambutol, and Vitamin B6.

Due to the epidemiological risk, spirometry was not initially performed. Nonetheless, maintenance treatment for COPD with bronchodilators (Indacaterol + Glycopyrronium and Salbutamol as needed) was continued. In addition, the patient received a short 10-day course of Prednisone at a dose of 0.5 mg/kg body weight, along with gastroprotection.

**Figure 3:**
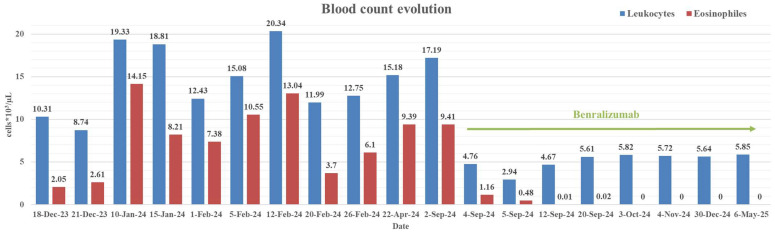
CBC evolution from 17 December 2024 to 6 May 2025, showcasing the impact of biologic therapy with benralizumab (started on 3 September 2024).

Initial laboratory investigations in December 2023 revealed marked eosinophilia, with an absolute eosinophil count of 2050/μL (19.95%), which persisted over the following months with oscillating but generally high values, peaking at 14,150/μL (73.2%) (see Figure 3).

In the effort to determine the etiology of the marked HE, extensive biological testing was conducted. These tests ruled out parasitic intestinal, fungal and viral infections (including HIV), vasculitis, connective tissue diseases, and sarcoidosis. A chest CT scan performed on 17 January 2024, revealed extensive and progressive infiltrative-nodular opacities, predominantly in the apical region of the right lung, with slight topographical changes compared to the previous scan. Bilateral pulmonary emphysema was also confirmed.

Subsequently, bronchoscopy and gastroenterological and urological investigations were performed to exclude neoplastic disease with thoracic, gastrointestinal, or genitourinary localization. The possibility of cerebral and ocular dissemination of parasitic infection was considered. Consequently, a non-contrast brain CT scan and a neurological consultation were performed, both of which revealed no evidence of acute focal cerebral lesions. Surprisingly, laboratory results showed a markedly elevated total IgE level of 2268.1 IU/mL (normal ≤ 158 IU/mL) and extremely high anti-*Toxocara canis* IgG antibody titers, with an index of 3892 (normal < 0.900).

Upon revisiting the patient history, the patient recalled a very relevant exposure: a two-month period working as a caretaker at a municipal dog shelter (housing approximately 200 dogs), as part of a social work program organized by the local social services. Based on these findings and history, a diagnosis of toxocariasis was established.

Antiparasitic therapy was added to the existing treatment regimen, starting with a 7-day course of Albendazole 400 mg twice daily. Due to persistent respiratory symptoms and ongoing HE, a second course of treatment was initiated after a 7-day interval, consisting of a 10-day regimen of Metronidazole 500 mg three times daily, targeting possible protozoal co-infections such as giardiasis and trichomoniasis. This was administered alongside Prednisone at 0.5 mg/kg body weight, with only partial clinical and laboratory improvement.

Seven days later, given the increase in eosinophil count after the 2nd course of antiparasitic treatment with Metronidazole (see Figure 3), when we were not yet thinking of HES but rather of a severe unresolved parasitic infection, a third course of antiparasitic treatment with Albendazole was initiated for 21 days with the same dosage. The patient tolerated all therapies well, including antituberculosis and antiparasitic medications, and experienced a partial clinical improvement.

**Figure 4:**
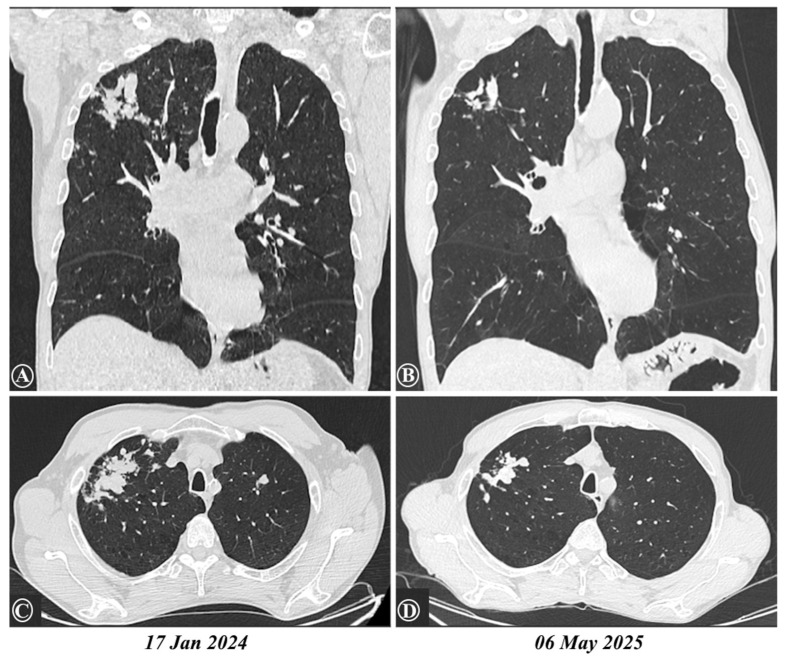
Comparative chest CTs from 17 January 2024 to 6 May 2025, with (**A**,**B**) presenting a coronal view and (**C**,**D**) presenting an axial section of the upper lobes, showed a reduction in the size of previous lesions, now appearing as fibro-nodular sequelae in the right lung apex, along with bilateral emphysema.

Despite repeated interventions, the HES remained persistent, with only transient decreases during periods of systemic corticosteroid therapy. Serologic monitoring revealed a persistent high level in both total IgE and *Toxocara* specific IgG antibodies over time (Table 1). Serial chest CT scans demonstrated favorable radiological evolution, with a reduction in the size of pulmonary lesions (Figure 4).

Unexpectedly, bacteriological investigations for tuberculosis, including cultures on both solid and liquid media, returned negative results at baseline, as well as at the 2-month and 6-month marks. These findings cast doubt on the initial diagnosis of active pulmonary tuberculosis, which had been made especially on the basis of a positive GeneXpert result and suggestive evolutive imaging findings. Nonetheless, standard antituberculosis therapy was continued for the full 6-month course, also supported by radiological reduction in the size of previous lung lesions. Concurrently, COPD management was maintained and augmented with the addition of a high-dose inhaled corticosteroid.

The clinical course remained fluctuating, with recurring respiratory exacerbations characterized by persistent dry cough, episodes of wheezing, and bilateral bronchial rales. Laboratory tests consistently showed persistent HES. Considering this, serum tryptase levels were measured and found to be elevated (46.1 µg/L; reference range < 11.4 µg/L), raising the suspicion of systemic mastocytosis associated with HES. This prompted further diagnostic workup and exploration of alternative therapeutic options.

Surprisingly, two spirometry tests performed 30 days apart revealed significant bronchodilator responsiveness: increases in FEV1 of 19% (+220 mL) and of 17% (+210 mL), respectively, following the administration of 400 µg of salbutamol. These findings supported a new diagnosis of severe, late-onset asthma, overlapping with COPD and HES. No signs of right heart failure were present [22].

By June 2024, the six-month course of antituberculosis treatment had concluded. However, both respiratory symptoms and HE persisted despite three courses of antiparasitic therapy and systemic corticosteroids. Further efforts led to successful genetic testing as recommended by hematology. Molecular cytogenetic analysis (FISH) for PDGFRA, PDGFRB, and FIP1L1 rearrangements returned negative, effectively ruling out an underlying myeloproliferative neoplasm.

Under these circumstances, the introduction of biological therapy targeting interleukin-5 receptor alpha (IL-5Rα) was pursued. This approach was strongly supported by GINA 2022 guidelines, due to the dual indications: severe, late-onset, treatment-refractory asthma (uncontrolled on maximal inhaled therapy with three systemic corticosteroid courses within six months) and persistent HES. Thus, on 3 September 2024, treatment with Benralizumab 30 mg subcutaneously was initiated, the first three doses administered every four weeks, then one dose every eight weeks. At the time, this was the only available biologic option recommended by current GINA guidelines and supported by the limited existing literature [23,24,25,26,27,28].

The therapeutic response was remarkable: eosinophil count dropped rapidly from 9410/μL to zero within 10 days (Figure 3). Over the following 10 months, the patient’s clinical, biological, and radiologic parameters remained stable and favorable, with no eosinophilic resurgence and no respiratory exacerbations. No adverse reactions to biologic therapy were reported.

## 3. Discussion

Tuberculosis and parasitic infections frequently coexist in many parts of the world, yet their interaction remains poorly understood. Tuberculosis is typically associated with type 1 immunity, characterized by intense phagocytic activity stimulated by activated type 1 helper T lymphocytes (Th1) that secrete interferon-gamma (IFN-γ), interleukin-2 (IL-2), and tumor necrosis factor-alpha (TNF-α), contributing to the formation of granulomas, structures that isolate the bacilli and limit their spread. IFN-γ activates macrophages to kill intracellular bacilli, and macrophages secrete interleukin-12 (IL-12), which promotes the differentiation of CD4+ T lymphocytes into Th1 cells. In contrast, in toxocariasis, Th2 cells are involved; they secrete IL-4, IL-5, IL-9, IL-10, and IL-13 and promote type 2 immunity, characterized by high antibody titers. While Th1 cells also contribute to antibody production, Th2 responses can antagonize Th1-mediated mechanisms. Th2 dominance can worsen tuberculosis, as antibodies are ineffective against intracellular pathogens. In general, for most infections, except those caused by large eukaryotic parasites, type 1 immunity is protective, while type 2 responses may help regulate or resolve excessive inflammation. However, in immunocompromised individuals or in the case of overwhelming microbial loads, the immune system may shift toward a type 2 response, even in infections typically controlled by type 1 immunity [29].

However, the extent to which a concurrent type 2 immune response, induced by parasitic infections, might impair the host’s ability to control mycobacterial infection is not clearly defined, nor are its direct effects on the progression of tuberculosis [30,31,32,33,34].

Our case is clinically significant as it illustrates the co-occurrence of several common conditions encountered in daily medical practice such as COPD, asthma, and tuberculosis, with a parasitic infection that is usually considered benign and often neglected. In this particular scenario, the combined presence of these conditions triggered a rare and dangerous complication: persistent severe hypereosinophilia.

Multiple differential diagnoses were considered in the evaluation of HE. A complex diagnostic workup was required to successively rule out other infectious etiologies, hematologic malignancies, vasculitis, paraneoplastic syndromes and systemic mastocytosis. Tryptase levels were elevated in this case, prompting further investigation into the possibility of mastocytosis. Unlike HES, which is more commonly seen in males, mastocytosis occurs with equal sex distribution. However, the eosinophil-to-tryptase ratio (AEC/tryptase) has been proposed as a useful marker to differentiate between the two conditions: a ratio below 100 typically favors systemic mastocytosis with eosinophilia, while a ratio above 100 is more consistent with HES [31]. In this case, the calculated ratio was 10,000/46.1 = 216.91, strongly supporting the diagnosis of HES.

The primary method for diagnosing human toxocariasis is serologic testing. However, determining whether an infection is recent remains challenging. Anti-*Toxocara* IgG antibodies can persist in the bloodstream for extended periods, making it difficult to distinguish between past and current infections. The most commonly used serological test for the routine diagnosis of *Toxocara canis* infection is the Enzyme-Linked Immunosorbent Assay (ELISA). This is highly sensitive and specific and detects IgG antibodies against antigens secreted by *Toxocara* larvae (TES—*Toxocara* Excretory-Secretory antigens). There is also a specialized ELISA that detects IgA anti-TES, but it is not routinely available, being used only in specialized research. In addition, there are studies that show that even IgA-anti TES values can persist up to 9 months after acute infection [35], so it is difficult to confirm acute infection even with this test. A 2022 study suggests that the low-avidity IgG test may serve as a more accurate marker for recent *Toxocara* infection [35], however unfortunately it is not available in our country’s healthcare plan.

Genetic testing played a critical role in establishing the final diagnosis by excluding an underlying hematologic malignancy [36]. The diagnosis of pulmonary tuberculosis was supported by multiple factors: the patient belonged to a high-risk group, resided in an endemic area, presented with clinical symptoms, had highly suggestive pulmonary lesions, and showed conversion of the GeneXpert test from negative in January 2023 to “low detected” in December 2023. The presence of apical fibronodular lesions in the right upper lobe in January 2023, with a positive Quantiferon TB Gold Plus test, in the absence of antituberculosis treatment, supports the passage through an older form of asymptomatic tuberculosis, which healed spontaneously, followed very likely by the recent reactivation of dormant bacilli, but with a reduced bacterial load.

However, the persistently negative bacteriological tests for *Mycobacterium tuberculosis* across multiple samples raised the possibility of a false-positive GeneXpert result which largely depends on the clinical context and the population tested. A meta analiysis of 187 fourfold tables from 72 studies revealed that GeneXpert MTB/RIF Ultra demonstrated an overall pooled sensitivity of 76% and specificity of 95% for detecting pulmonary tuberculosis [37]. False-positive results can occur in the case of residual bacterial DNA (which can persist after complete treatment or after spontaneous recovery, even if the bacilli are dead), or due to laboratory settings and quality errors. On the other hand, if the bacterial load is very low, classic examinations, namely direct microscopy and cultures, can be negative. Nevertheless, in our case, the most likely scenario is the endogenous reactivation of dormant bacilli within pre-existing apical Ghon foci in the right lung.

It is worth noting that tuberculosis has been associated with HE, particularly in pediatric populations [38,39,40]. Additionally, any medication—including antituberculosis drugs—can induce drug hypersensitivity reactions or drug-associated eosinophilia. However, in this case, the presence of HE before initiation of tuberculosis treatment, and its persistence after treatment cessation, ruled out drug-induced eosinophilia.

Severe HE represented the central diagnostic and therapeutic challenge in this case. Several therapeutic interventions carried potential risks, including prolonged antiparasitic treatment administered concomitantly with antituberculosis medication, repeated systemic corticosteroid use, and ultimately the initiation of biologic therapy with benralizumab in a parasitic infection. The confirmation of severe asthma in this HES context supported our consideration of benralizumab as an eosinophil-depleting treatment, in accordance with the recommendations of the GINA 2022 guidelines. All interventions were performed with the patient’s informed consent and under close hospital supervision.

The underlying immunological rationale was that parasitic infections generally elicit a type 2 immune response in the host, and eosinophils modulate inflammatory responses involved in tissue remodeling, repair, parasite defense, and allergic reactions [6].

Benralizumab is a humanized, afucosylated IgG1 kappa monoclonal antibody targeting the alpha subunit of the interleukin-5 receptor (IL-5Rα), which is specifically expressed on eosinophils and basophils. Its afucosylated Fc domain enhances affinity for FcγRIII receptors on effector immune cells such as natural killer (NK) cells, leading to apoptosis of eosinophils and basophils via antibody-dependent cell-mediated cytotoxicity (ADCC). This distinct mechanism sets benralizumab apart from other anti-IL-5 agents, enabling effective eosinophil depletion and suppression of eosinophilic inflammation, a response clearly observed in our case through the rapid and sustained resolution of both eosinophilia and clinical manifestations [41].

Given the diagnostic and therapeutic complexity of our case and the scarcity of available data on similar clinical scenarios, we conducted a literature review focused on publications from the last ten years indexed in Clarivate, PubMed, ResearchGate, and Google Scholar. The first aim was to identify case reports describing similar diagnostic challenges, while the second aim focused on reports involving overlapping complications, comorbidities, and therapeutic strategies.

Using five key search terms: toxocariasis, tuberculosis, hypereosinophilia, asthma and benralizumab, we found no relevant case reports or studies published in the last decade. Also, a similar outcome was observed when limiting the search to four terms: toxocariasis, tuberculosis, hypereosinophilia and asthma. However, when the search was narrowed to three keywords, toxocariasis, tuberculosis, hypereosinophilia, two articles were retrieved from PubMed (published in 2021 [42] and 2022 [43]). Although not directly comparable to our case, both underscore the importance of active screening for tuberculosis and parasitic infections, especially in vulnerable populations.

The first article, a 2021 cross-sectional study conducted in southeastern Iran, evaluated the seroprevalence of toxocariasis in 373 children aged 3–13 years. The study reported a seroprevalence rate of 1.3%, with statistically significant associations between infection and eosinophilia, contact with dogs or cats, and consumption of raw vegetables or contaminated water [44].

The second article, from 2022, examined the impact of the COVID-19 pandemic on screening for tuberculosis and other imported diseases among migrant minors in Spain. This retrospective cross-sectional study included 192 children, most of whom were asymptomatic. Eosinophilia was observed in 20.8% of cases, with the most common diagnoses being latent tuberculosis infection (72.9%), schistosomiasis (15.1%), toxocariasis (4.9%), and strongyloidiasis (4.9%) [45].

Despite the lack of directly comparable case reports, the combination of *Toxocara* infection, *Mycobacterium tuberculosis*, and HE has been broadly addressed in medical literature. A search on ResearchGate yielded 2248 articles, and Google Scholar identified 330 relevant publications from the past decade. These studies consistently confirm the high prevalence of these conditions individually and, in some instances, their coexistence [32,33,34].

A 2024 review article explored the immunological interactions and natural resistance mechanisms involved in tuberculosis, toxocariasis, and their coexistence. It concluded that while each condition elicits distinct immune responses, their interaction may significantly alter host immunological reactivity. The authors highlighted that the immune dynamics in patients simultaneously affected by tuberculosis and toxocariasis remain insufficiently understood and require further investigation [34].

HE has become an area of growing clinical interest due to its increasing incidence and prevalence [14,16,19], expanding understanding of its underlying pathophysiological mechanisms [5,6,8,9,19], and the emergence of novel biologic and targeted oncologic therapies. Recent publications include updated HE diagnostic and treatment guidelines [11,12,13], position statements from national medical societies, such as the 2024 publication by the Italian Society of Allergology, Asthma and Clinical Immunology [44] and expert recommendations advocating for a precision medicine approach based on molecular diagnostics [46,47,48,49]. Most patients with HE who are treated with current biologic therapies, including benralizumab, have shown favorable responses with minimal adverse effects [28,29,49].

Our case stands out for several reasons. It involves an unusual and unfavorable association between *Toxocara canis* infestation and tuberculosis, which triggered secondary complications such as severe reactive HE and late-onset asthma. Diagnostic clarity was hindered by overlapping clinical and radiological features between toxocariasis and tuberculosis, as well as between asthma and COPD. The therapeutic course was also unconventional, with benralizumab—an anti-eosinophilic biologic agent—used successfully after a parasitic infection.

Despite involving infections that are commonly encountered in clinical practice, we found no similar case in the reviewed literature that described the combination of toxocariasis and tuberculosis leading to severe, persistent HE and the emergence of late-onset severe asthma. Furthermore, we identified no previous reports in which benralizumab was successfully used in a parasitic infection context with such clearly positive outcomes.

This case has several important clinical implications. First, it emphasizes the need to investigate even seemingly minor parasitic infections in adults presenting with eosinophilia and chronic cough, particularly in the context of severe, uncontrolled asthma. This is especially crucial in individuals with known risk factors for impaired immune response, such as smoking, alcohol use, or ongoing immunosuppressive therapy.

One of the primary diagnostic challenges lies in determining the chronicity of the parasitic infection is whether the infection is acute or represents residual immune memory and in choosing the appropriate treatment strategy. In cases where antiparasitic therapy fails to resolve reactive HE driven by a type 2 immune response, and clinical and imaging findings remain unchanged, biologic therapy may be a valid and effective option, as demonstrated in our case.

From a practical standpoint, minimizing diagnostic delay and ensuring timely initiation of appropriate therapy are critical, especially given the significant impact of HE on quality of life and its potential to cause or exacerbate other complications.

The main limitations of our research are as follows, we were not able to determine specific biomarkers (such as IL-2, IFN-γ, CD45RA-, CCR6-, CCR4-, CXCR3+, CCR5+, etc.) to assess the magnitude and ratio between the type 1 and type 2 immune responses, we can not know for certain the onset and duration of the two co-infections, and we performed a limited review of the specialized literature (our search was limited to four databases). Consequently, while we were unable to identify comparable cases of toxocariasis and tuberculosis associated with severe comorbidities and successful biologic therapy, our findings cannot be generalized.

## 4. Materials and Methods

To identify scientific articles presenting clinical cases similar to our patient, specifically those involving the coexistence of toxocariasis, tuberculosis, HE, and asthma treated with benralizumab, we conducted a targeted literature search across four major international databases: Clarivate, PubMed, ResearchGate and Google Scholar. The search was limited to articles published in English over the past ten years. Each database was queried individually using the following set of keywords: “toxocariasis”, “tuberculosis”, “hypereosinophilia”, “asthma”, and “benralizumab”. The search algorithm for each database was developed using a variation in the following Clarivate query: “toxocariasis (All Fields) AND tuberculosis (All Fields) AND hypereosinophilia (All Fields) AND asthma (All Fields) AND benralizumab (All Fields).” Because preliminary searches restricted to specific fields (title or topic) yielded no results, the search was broadened to include all fields to maximize retrieval of any potential matches. Additionally, equivalent or related terms (such as *Toxocara*, HES, eosinophilia) were tested as alternative keywords, however, these attempts likewise produced no relevant results. Following these results, we decreased the number of search terms by removing benralizumab and following the same search pattern, this attempt yielded no meaningful results as well.

Despite extensive research, we did not identify any published case reports describing a clinical scenario fully comparable to ours. However, we found numerous articles and a few review papers discussing toxocariasis, tuberculosis, and, in some cases, their coexistence with secondary eosinophilia. Additionally, there is a substantial body of literature validating the efficacy of biologic therapies in the management of HE, including therapies targeting the IL-5 pathway such as benralizumab.

That said, no reports were identified in which biologic therapy was initiated in a patient with a confirmed parasitic infection, likely due to prevailing concerns that immunomodulatory treatment could exacerbate or reactivate parasitic infections.

## 5. Conclusions

Concomitant *Toxocara* and tuberculosis infection induces a broad spectrum of immune system alterations through both type 1 and type 2 immune mechanisms and manifests as severe, persistent hypereosinophilia and asthma. Paradoxically, in our case, biological therapy with benralizumab proves to be a highly effective intervention.

## Figures and Tables

**Table 1:** Paraclinical parameters monitored between 12 January 2023 and 6 May 2025, which show the evolution of systemic inflammation, respiratory function and a clear therapeutic response to benralizumab.

Parameter (Normal Value)	12 January 2023	18 December 2023	3 September 2024	6 May 2025
BMI (kg/m^2^)	20.9	19.59	21.22	21.79
FEV1 (normal estimated 3.5 L)	1.82 (52%)	-	1.5 ^a^ (43%)	1.86 ^a^ (53%)
CRP (0–5 mg/L)	6.12	9.11	32.5	1.14
ESR (3–10 mm/1 h)	15	10	20	5
Leucocytes (4–10 × 10^3^/µL)	6.2	10.31	17.19	5.85
Eosinophils (0–0.7 × 10^3^/µL)	0.26 (3.7%)	2.61 (29.9%)	9.41 (54.7%)	0 (0%)
Hemoglobin (14–17.2 g/dL)	12.4	14.1	13.5	16
IgE (0–100 UI/mL)	-	2268.1	13,480.2	>2500 ^b^
*Toxocara canis* IgG (index < 0.9)	-	-	3.892	4.168

a: Post-bronchodilator therapy; b: the testing method used does not measure values above 2500 UI/mL.

## Data Availability

The datasets are not publicly available. Anonymized data may be provided upon request from Dr. Nicoleta Bertici.

## Abbreviations

The following abbreviations are used in this manuscript:

AEC	Absolute Eosinophil Count
ADCC	Antibody-Dependent Cell-mediated Cytotoxicity
BMI	Body Mass Index
CBC	Complete Blood Count
CI	Confidence Interval
COPD	Chronic Obstructive Pulmonary Disease
CRP	C-Reactive Protein
CT	Computed Tomography
ELISA	Enzyme Linked Immunosorbent Assey
ESR	Erythrocyte Sedimentation Rate
FEV1	Forced Expiratory Volume in 1 s
FIP1L1	Factor Interacting with PAPOLA and CPSF1
FISH	Fluorescence In Situ Hybridization
GINA	Global Initiative for Asthma
HE	Hypereosinophilia
HES	Hypereosinophilic Syndrome
IL	Interleukine
LABA	Long-acting beta-agonists
LAMA	Long-Acting Muscarinic Antagonists
NK	Natural Killer cells
PDGFRA	Platelet-Derived Growth Factor Receptor Alpha
PDGFRB	Platelet-Derived Growth Factor Receptor Beta
Th1	T-helper 1 cells
Th2	T-helper 2 cells
TNF-α	Tumor Necrosis Factor-alpha
WHO	World Health Organisation
